# Validation of the Arabic Version of the Multicultural Quality of Life Index (MQLI-Ar) Among Parents of Children with Disabilities

**DOI:** 10.3390/healthcare14101327

**Published:** 2026-05-13

**Authors:** Majed Ahmed Algarni, Abdullah Ahmed Alghamdi, Mohammad S. Alzahrani

**Affiliations:** 1 Department of Clinical Pharmacy, College of Pharmacy, Taif University, P.O. Box 11099, Taif 21944, Saudi Arabia; m.s.alzahrani@tu.edu.sa; 2 Department of Special Education, College of Education, Taif University, P.O. Box 11099, Taif 21944, Saudi Arabia; abdullh@tu.edu.sa

**Keywords:** caregiver burden, children with disabilities, cross-sectional studies, factor analysis, statistical, psychometrics, quality of life, validation studies

## Abstract

**Background:** Parents of children with disabilities often experience reduced quality of life (QoL), yet validated Arabic instruments remain limited. This study aimed to translate and validate the Arabic version of the Multicultural Quality of Life Index (MQLI-Ar). **Methods:** A cross-sectional study was conducted among 132 parents and caregivers in Saudi Arabia. The MQLI was translated using forward–backward translation and culturally adapted. Reliability and validity were assessed using Cronbach’s alpha, exploratory factor analysis (EFA), and confirmatory factor analysis (CFA). **Results:** The MQLI-Ar demonstrated high internal consistency (Cronbach’s alpha = 0.948). EFA supported a unidimensional structure explaining 68.3% of the variance. CFA indicated acceptable model fit (CFI = 0.934, TLI = 0.915, SRMR = 0.0397), although RMSEA was elevated. Factor loadings ranged from 0.642 to 0.919, confirming strong item–factor relationships. **Conclusions:** The MQLI-Ar is a reliable and valid tool for assessing QoL among Arabic-speaking caregivers of children with disabilities, supporting its use in research and clinical practice.

## 1. Introduction

The recent statistics have indicated that the prevalence of disabilities in Saudi Arabia is approximately 5.9%. This notably significant prevalence translates into 1,349,585 individuals within a total population of 32,175,224 [1]. Disability in Saudi Arabia is legally acknowledged, and the government offers a wide array of services to support individuals with disabilities. These services incorporate various categories of disabilities, including learning disabilities (LD), autism, attention deficit and hyperactivity disorder (ADHD), hearing impairments, and other types of disability [2]. Given the substantial number of individuals with disabilities and the diversity of disability types, the government established the Authority of People with Disabilities in 2018. The purpose of this authority is to address the needs of individuals with disabilities and collaborate with other governmental sectors, such as the ministries of education, health, and social affairs. The Authority also engages with international organizations dedicated to supporting individuals with disabilities [3].

Depending on the specific characteristics and its severity, any disability can significantly impact the quality of life (QoL) in multiple dimensions. Caregivers, particularly parents of children with disabilities, encounter numerous challenges and barriers that affect their own lives in diverse ways. These challenges may include time limits, social pressures in certain cultures, and financial difficulties [4].

The overall QoL of individuals is shaped by various factors, such as socioeconomic status (SES), access to education, health conditions, family relationships, and other relevant aspects. However, having a child with a disability can profoundly transform family dynamics and the QoL, which further depends on the nature of the disability, its severity, and the breadth of its impact [5].

The most direct effect of having a child with disability is observed within the family unit, particularly affecting the father, mother, and sibling. This is primarily due to their direct responsibility for the child’s upbringing, monitoring their condition, and overseeing their educational development, as well as addressing all related personal and medical needs. Since the burden of care and support largely falls upon the family, parents play a crucial role in providing care for their child with a disability [6]. Therefore, family support is strongly linked to improving the child’s health and psychosocial outcomes [7]. However, several studies have documented that having children with disability such as autism or intellectual disabilities negatively affects the parents’ QoL and their psychological well-being [8,9,10].

Despite studies highlighting the effects of a child’s disability on parents, research on disabilities has mainly focused on the growth of children, teens, and adults, leaving the quality of life for parents largely unexamined [11]. Furthermore, the effects of a care recipient’s disorder on caregivers are often examined through frameworks such as caregiver burden and parenting stress [12,13]. Galloway et al. argued that parents’ perceived psychological well-being and stress levels can significantly influence their child’s QoL [14]. Therefore, interventions aimed at alleviating parental stress and enhancing their QoL may lead to improvements in the QoL of their children as well.

Although QoL of parents of children with disability has gained more attention, and several studies have compared QoL of parents of children with disability to QoL of parents of typically general population norms [12,15,16,17], there remains a deficiency of validated instruments to assess QoL for this group particularly in Arabic language. QoL serves as an essential outcome measure in directing healthcare as it is viewed as a significant factor in assessing healthcare effectiveness [18,19].

The concept of QoL can be articulated in various ways, emphasizing an optimal state characterized by overall well-being, where an individual’s daily functioning across multiple domains is affected by the potentially negative consequences of illness or disorder [20]. Quality of life can be conceptualized as either health-related QoL (HRQoL), focusing on physical and functional health outcomes, or broader subjective QoL encompassing psychological, social, and existential domains [21,22]. Instruments such as the WHOQOL-BREF and SF-36 emphasize health status and functional limitations [23], whereas the Multicultural Quality of Life Index (MQLI) provides a concise, multidimensional assessment incorporating subjective well-being, including spiritual and existential domains [24].

Despite the existence of numerous measures aimed at assessing QoL, there are relatively few concise self-reported questionnaires that encompass functioning, social interactions, and environmental factors [25,26]. Furthermore, there is limited evidence regarding the effectiveness of scales that evaluate QoL, specifically among parents of children with disabilities. Therefore, the brief generic Multicultural Quality of Life Index (MQLI) was created to assess health-related QoL across diverse cultures, drawing from a comprehensive review of global literature. It includes 10 dimensions of subjective QoL, addressing areas from physical health to spiritual well-being, along with an overall assessment of QoL [24]. The MQLI, now offered in six languages—English, Spanish, German, Chinese, Korean, and Greek—is a culturally aware instrument as it has been validated among diverse populations and through different factor analysis methods [27,28,29,30,31].

Parents and caregivers of children with disabilities often face greater challenges than those of typically developing children, as raising and supporting them demands additional physical, financial, and emotional effort. By translating the MQLI tool into Arabic language, we aim to enable researchers in Saudi Arabia and other Arabic-speaking countries to explore QoL and its impact on various aspects and conditions by using a reliable, efficient, and simple tool.

The present study aimed to translate and culturally adapt the Multicultural Quality of Life Index (MQLI) into Arabic and evaluate its psychometric properties among parents of children with disabilities. Specifically, the study sought to assess internal consistency reliability, examine construct validity using exploratory and confirmatory factor analysis, and describe quality of life levels in the target population.

## 2. Methods

### 2.1. Study Design and Setting

This study was conducted as a cross-sectional survey utilizing a standardized, self-reported, web-based questionnaire. Prior to participation, informed consent was obtained from each participant. Data were collected between January 2025 and March 2025. Participants were recruited through social media platforms, caregiver support groups, and healthcare centers, using a convenience sampling approach. Inclusion criteria included Arabic-speaking parents or primary caregivers aged 18 years or older, residing in Saudi Arabia, and caring for a child with a formally diagnosed disability. Exclusion criteria included individuals who were not primary caregivers or those unable to complete the questionnaire in Arabic. All data were collected anonymously and stored in a secure, password-protected database accessible only to the research team. No identifiable information was recorded, ensuring confidentiality and compliance with ethical research standards. The study protocol was reviewed and approved by the Institutional Review Board (IRB) of Taif University (approval no. 45-128). Participants were informed about the study objectives and their right to withdraw at any time before providing consent.

### 2.2. Survey Tool

The survey employed in this study comprised demographic questions and the items of the MQLI. The demographic section included questions regarding the respondent’s age, gender, educational level, income, and region of residence, as well as the age and disability diagnosis of their child.

The MQLI consists of 10 items, each designed to systematically assess distinct dimensions of quality of life. These domains include physical health, psychological well-being, autonomy in daily activities, occupational performance, interpersonal relationships, emotional support networks, access to community resources, personal satisfaction, spiritual fulfillment, and an overall evaluation of life quality. Responses are recorded on a 10-point scale, with higher scores indicating a more favorable assessment of quality of life in the respective domain. The cumulative score ranges from 10 to 100, with higher values reflecting an enhanced overall quality of life.

To ensure the accuracy and cultural relevance of the MQLI-Ar for Arabic-speaking participants, we followed established cross-cultural translation guidelines [32]. The translation and cross-cultural adaptation process followed standardized methodological guidelines. The original English version of the MQLI was independently translated into Arabic by two bilingual experts with experience in healthcare research (both fluent in English and Arabic). A third independent translator, blinded to the original instrument, performed back-translation into English to ensure conceptual equivalence between the original and translated versions.

The pre-final Arabic version was pilot tested on a small sample of participants representative of the target population (n = 15) to assess clarity, comprehensibility, and cultural appropriateness of the items. Feedback from the pilot participants indicated minor linguistic ambiguities in certain items, particularly in items related to personal fulfillment and community/services support, which were subsequently refined to improve clarity.

Content validity was further evaluated by an expert panel consisting of three specialists in clinical pharmacy, psychometrics, and public health. Each expert independently assessed the relevance and clarity of the items using a qualitative evaluation approach. Minor modifications were made based on expert feedback to enhance linguistic clarity and contextual suitability. As this phase focused on qualitative expert evaluation during cross-cultural adaptation, content validity was established through expert consensus in accordance with established guidelines [33], rather than calculation of a formal Content Validity Index (CVI).

**Figure 1:**
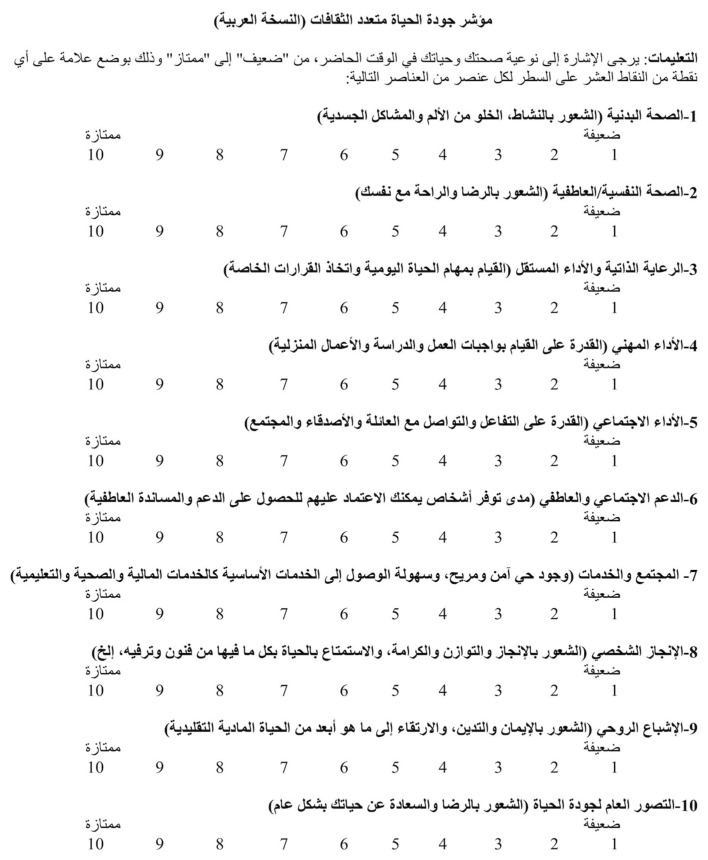
The Arabic translation of the Multicultural Quality of Life Index (MQLI-Ar).

The Arabic translation of this survey tool is presented in Figure 1.

### 2.3. Statistical Analysis

Descriptive statistics, including frequencies, percentages, means, and standard deviations, were calculated to summarize the demographic and clinical characteristics of the respondents. The internal consistency reliability of MQLI-Ar was assessed using Cronbach’s alpha coefficient. Corrected item-total correlations were calculated to evaluate the relationship between each item and the overall scale. The mean and standard deviation for each item were reported, along with Cronbach’s alpha if each item was deleted, to determine the impact of individual items on the internal consistency of the scale.

Exploratory Factor Analysis (EFA) with principal component extraction was performed to examine the factor structure of the MQLI-Ar. The eigenvalue criterion (>1) and the scree plot were used to determine the number of factors to retain. The Kaiser-Meyer-Olkin (KMO) measure of sampling adequacy and Bartlett’s test of sphericity were employed to assess the suitability of the data for factor analysis. Factor loadings were examined to identify the items contributing significantly to the underlying construct.

A Confirmatory Factor Analysis (CFA) was subsequently performed to validate the factor structure identified in the EFA and to align with recommended practices in cross-cultural scale validation. Model fit was evaluated using multiple indices, including the chi-square statistic (χ^2^), Comparative Fit Index (CFI), Tucker–Lewis Index (TLI), Root Mean Square Error of Approximation (RMSEA) with 90% confidence interval, and Standardized Root Mean Square Residual (SRMR). Standardized factor loadings and item communalities (R^2^) were examined to assess the strength of item–factor relationships.

All descriptive statistics, reliability analyses, and EFA procedures were conducted using SPSS (version 22.0). The CFA was performed using jamovi (version 2.6.44), which implements the lavaan structural equation modeling engine. Statistical significance was set at *p* < 0.05.

The sample size of 132 participants was deemed sufficient for conducting EFA, surpassing the frequently recommended 10:1 participant-to-item ratio, which has been widely supported in methodological research [34]. Additionally, the high KMO value and strong factor loadings further validate the adequacy of the sample.

## 3. Results

### 3.1. Demographic and Clinical Characteristics

The demographic and clinical characteristics of the respondents (N = 132) are summarized in Table 1. The majority of respondents were mothers (77.3%), followed by fathers (16.7%) and grandparents/siblings (6.1%). The age distribution of caregivers was as follows: 14.4% were under 30 years, 37.1% were between 30 and 40 years, 34.8% were between 40 and 50 years, and 13.6% were over 50 years. Regarding educational attainment, 52.3% of caregivers had completed high school or less, 39.4% held a bachelor’s degree, and 8.3% had higher education qualifications. In terms of income, the majority of respondents reported (43.2%) earning less than SR 6000. A fair percentage of respondents (59.1%) resided in the Makkah region. The ages of the children cared for by the respondents were distributed as follows: 21.2% were under 6 years, 43.9% were between 7 and 12 years, and 34.8% were over 13 years. The most frequently reported disability was autism (34.8%), followed by LD (25%), mental disability (24.2%), and ADHD (18.9%).

### 3.2. Reliability and Internal Consistency

The internal consistency reliability of the MQLI-Ar was found to be high, with a Cronbach’s alpha coefficient of 0.948. This indicates a high level of internal consistency among the 10 items on the scale, suggesting that the items reliably measure the same underlying construct of QoL. The mean scores, corrected item-total correlations, and Cronbach’s alpha if the item was deleted for the 10 items are presented in Table 2. The mean scores ranged from 6.69 (physical well-being) to 7.87 (global perception of QoL), indicating moderate to high levels of well-being among respondents. Corrected item-total correlations varied between 0.637 (physical well-being) and 0.890 (personal fulfillment), showing substantial consistency across items. The Cronbach’s alpha values if items were deleted ranged from 0.937 to 0.948, demonstrating high internal consistency for the scale as a whole. The highest corrected item-total correlations were notably observed for interpersonal functioning (0.866) and personal fulfillment (0.890), suggesting these items strongly correlated with the overall scale. The scale’s robustness is further supported by high reliability coefficients, highlighting its reliability in assessing various dimensions of respondents’ QoL.

### 3.3. Factor Analysis

**Figure 2:**
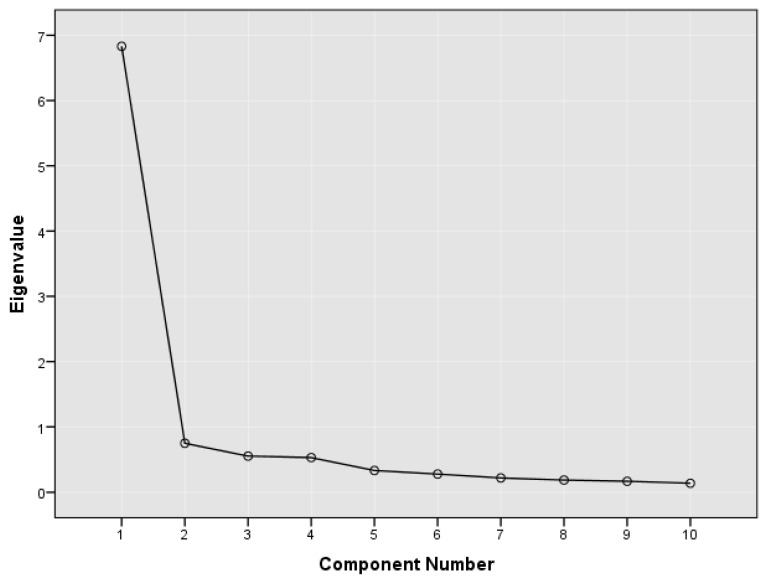
Scree Plot of Eigenvalues for Principal Component Analysis of the MQLI-Ar.

The eigenvalue criterion in our study indicated the extraction of a single factor. We conducted an unrotated EFA on the 10 items of the MQLI-Ar, which produced one component with eigenvalues greater than 1, adhering to KMO’s criterion [35]. This factor explained 68.3% of the total variance, underscoring the unidimensionality of the quality-of-life instrument. The scree plot corroborated this result, suggesting a one-factor solution at the inflection point (Figure 2). The KMO measure of sampling adequacy was 0.933, significantly exceeding the recommended threshold of 0.8, confirming the data’s appropriateness for EFA. Additionally, Bartlett’s test of sphericity was significant (*p* < 0.001), indicating that the correlations among items were sufficiently large to justify their inclusion in the EFA.

The factor loadings of the 10-item MQLI-Ar are shown in Table 3. All items had substantial factor loadings, indicating that they significantly contribute to the underlying construct being measured. The factor loadings ranged from 0.695 (physical well-being) to 0.917 (personal fulfillment), with the highest loading observed for personal fulfillment, suggesting it has the strongest association with the overall QoL. Other items with high factor loadings included interpersonal functioning (0.898), global perception of QoL (0.864), and social emotional support (0.858), indicating their significant impact on the QoL. The robust factor loadings across all items confirm the reliability and validity of the scale in assessing multiple dimensions of the QoL among respondents.

The one factor CFA model demonstrated acceptable to good fit on several indices. Although the chi-square test was significant (χ^2^(35) = 138, *p* < 0.001), this is expected given sample size sensitivity. The CFI = 0.934 and TLI = 0.915 indicated acceptable incremental fit, while the SRMR = 0.0397 reflected excellent absolute fit. The RMSEA = 0.131 (90% CI: 0.108–0.154) was elevated; however, this pattern is commonly observed in models with few degrees of freedom and strong factor loadings, and should be interpreted with caution alongside other fit indices [36].

**Figure 3:**
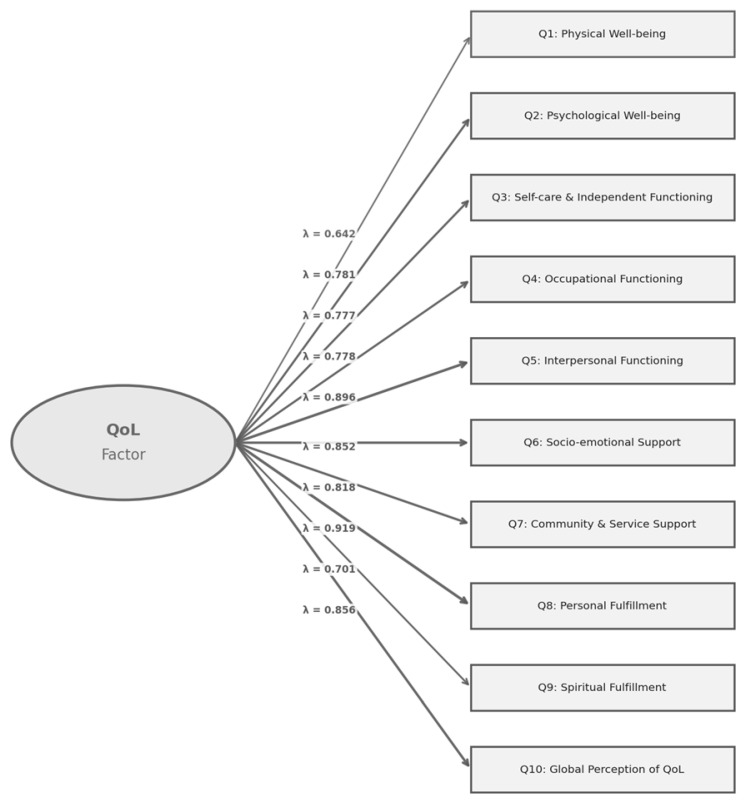
CFA Path Diagram of the Unidimensional MQLI-Ar Model. Rectangles represent observed item variables; the circle represents the latent QoL factor. Numbers on paths are standardized factor loadings (λ).

Standardized factor loadings were all substantial, ranging from 0.642 (physical well-being) to 0.919 (personal fulfillment) (Figure 3), confirming strong item–factor relationships. Items assessing interpersonal functioning (0.896), global perception of QoL (0.856), and socio-emotional support (0.852) demonstrated particularly high loadings. Communalities (R^2^) ranged from 0.413 to 0.844, indicating that the latent QoL factor explained a substantial proportion of variance across items.

## 4. Discussion

Parents and caregivers of children with disabilities commonly encounter more challenges compared to parents of typically developing children since raising and caring for such children requires extra physical, financial, and emotional effort and support [4,25]. Nonetheless, a reliable and culturally appropriate instrument is essential for accurately assessing QoL in this population. The present study contributes to this need by providing psychometric validation of the MQLI-Ar within an Arabic-speaking caregiver population.

The purpose of this study, therefore, was to examine the validity and reliability of the MQLI-Ar with parents and caregivers of children with disabilities who are Arabic speakers residing in Saudi Arabia. The findings provided valuable insights into the internal consistency, reliability, and factor structure of MQLI-Ar, as well as the self-reported QoL of the participants.

One intriguing finding of this study is that, in comparison to earlier research [24,28,37], the spiritual fulfillment factor is noticeably greater in MQLI-Ar. This may reflect cultural influences, as spiritual beliefs are strongly embedded within Saudi society; however, this interpretation remains exploratory and warrants further empirical investigation, particularly in studies examining religiosity and coping mechanisms among caregivers of children with disabilities.

Another notable finding is that the social and emotional support factor was higher than in previous studies [24,28,37] which may reflect strong social and family cohesion within Saudi society. Similarly, the community and services support factor was higher than reported in previous studies [24,28,37], which may indicate that the Saudi government is trying to provide special services to disability children and parents, especially when we know that many of the services and medications provided by the government are free of charge.

The MQLI-Ar demonstrated high internal consistency, with a Cronbach’s alpha of 0.948. This finding is higher than that reported in previous validation studies, such as the Norwegian validation by Mundal et al. [37], which reported an overall Cronbach’s α of 0.73 along with weaker item–total correlations for certain domains. In the present study, item–total correlations ranged from 0.637 to 0.890, indicating that all items contributed meaningfully to the overall scale. However, it is important to note that Cronbach’s alpha values approaching 0.95 may suggest potential item redundancy. Therefore, while the MQLI-Ar demonstrates strong internal consistency, future studies should further examine the dimensional distinctiveness of items to ensure that each domain contributes unique information to the assessment of quality of life.

The EFA revealed a unidimensional structure for the MQLI-Ar, with a single factor explaining 68.3% of the variance. This finding aligns with the original conceptualization of the MQLI as a measure of overall QoL rather than a multidimensional construct. The strong factor loadings across all items (ranging from 0.695 to 0.917) further affirm the robustness of the scale. Items such as personal fulfillment, interpersonal functioning, and global perception of QoL exhibited the highest loadings, reinforcing their critical importance in assessing overall QoL among caregivers.

The high KMO measure (0.933) and significant Bartlett’s test of sphericity confirm the suitability of the data for factor analysis. These results demonstrate that the MQLI-Ar retains its validity and reliability in assessing QoL in this Saudi cultural and Arabic context.

These findings are consistent with previous validation studies conducted in diverse cultural contexts, including Greek [28], Korean [31], Chinese [29], Spanish [30], Argentine [27], and Norwegian [37], all of which reported strong psychometric properties of the MQLI. This consistency reinforces the robustness and cross-cultural applicability of the instrument in assessing quality of life across different populations. This supports the generalizability of the MQLI-Ar beyond the current study population.

From a broader perspective, the strong psychometric performance of the MQLI-Ar suggests that the construct of quality of life, as measured by this instrument, is stable across different cultural contexts. This highlights the potential utility of the MQLI-Ar not only as a research tool but also as a practical instrument for clinical assessment and policy evaluation. In particular, the ability to capture multidimensional aspects of well-being—including psychological, social, and spiritual domains—makes it especially relevant for populations experiencing complex caregiving burdens, such as parents of children with disabilities.

A key strength of this study lies in the rigorous psychometric validation of the MQLI-Ar within a culturally specific population of parents and caregivers of children with disabilities. However, MQLI-Ar can generally be applicable to other Arabic parents’ speakers in other Arabic-speaking countries. The questionnaire demonstrated strong psychometric properties, reinforcing its reliability for use in this population. The inclusion of parents from different regions of Saudi Arabia enhances the generalizability of the findings. The sample size of this study was also adequate to validate the tool.

Several limitations should be acknowledged. First, the use of convenience sampling may limit the representativeness of the findings and introduce selection bias. Second, data collection through online platforms may have excluded individuals with limited digital access, resulting in potential digital access bias. Third, the sample was disproportionately drawn from the Makkah region, which may affect the generalizability of the results to other regions of Saudi Arabia. Fourth, the use of a self-reported questionnaire may introduce social desirability bias. Fifth, the absence of a comparison group (e.g., parents of children without disabilities) limits the ability to contextualize the findings. Sixth, the cross-sectional design precludes assessment of test–retest reliability and causal relationships. Finally, although CFA supported the one-factor structure, the use of the same sample for both EFA and CFA and the relatively modest sample size for structural equation modeling may limit the stability of model fit estimates; future studies with larger independent samples are recommended.

## 5. Conclusions

This study validated the MQLI-Ar among parents and caregivers of children with disabilities in Saudi Arabia. The findings demonstrated high internal consistency (Cronbach’s alpha = 0.948), although values approaching 0.95 may indicate potential item redundancy, alongside a unidimensional factor structure, supporting the reliability and construct validity of the instrument for assessing quality of life (QoL) in this population. The high corrected item–total correlations and substantial factor loadings further reinforce the robustness of the scale across multiple QoL domains, with interpersonal functioning and personal fulfillment emerging as key contributors.

The study highlights the applicability of the MQLI-Ar as a reliable and culturally appropriate tool for evaluating the well-being of Arabic-speaking caregivers. Its brevity and ease of administration, combined with strong psychometric performance, make it a practical instrument for both research and clinical settings. Notably, domains related to social and emotional support, community and services support, and spiritual fulfillment showed relatively higher scores compared to findings reported in previous studies, suggesting potential contextual or cultural influences.

Overall, the MQLI-Ar provides a scientifically sound and practical measure for assessing QoL among Arabic-speaking populations and offers valuable insights to inform targeted interventions and support programs for caregivers of children with disabilities.

## Figures and Tables

**Table 1:** Demographic and Clinical Characteristics of Respondents (N = 132).

Characteristics	Frequency (%)
Respondent	
Mother	102 (77.3)
Father	22 (16.7)
Grandparent/sibling	8 (6.1)
Age of caregiver	
<30 years	19 (14.4)
30–40 years	49 (37.1)
40–50 years	46 (34.8)
>50 years	18 (13.6)
Education level of caregiver	
High school (or less)/diploma	69 (52.3)
Bachelor’s degree	52 (39.4)
Higher education	11 (8.3)
Income	
<SR 6000	57 (43.2)
SR 6000–SR 12,000	44 (33.3)
>SR 12,000	31 (23.5)
Region	
Makkah region	78 (59.1)
Others	54 (40.9)
Age of child	
<6 years	28 (21.2)
7–12 years	58 (43.9)
>13 years	46 (34.8)
Most frequently reported disability *	
Autism	46 (34.8)
Learning disability	33 (25)
Mental disability	32 (24.2)
ADHD	25 (18.9)
Physical disability	8 (6.06)
Hearing disability	5 (3.79)
Visual disability	5 (3.79)

* Some children are diagnosed with more than one disability.

**Table 2:** Reliability and Internal Consistency of the MQLI-Ar.

Item	Mean ± SD	Corrected Item-Total Correlation	Cronbach’s Alpha if Item Deleted
Physical well-being	6.69 ± 2.74	0.637	0.948
2. Psychological/emotional well-being	7.29 ± 2.86	0.753	0.943
3. Self-care and independent functioning	7.67 ± 2.70	0.780	0.942
4. Occupational functioning	6.98 ± 2.94	0.767	0.943
5. Interpersonal functioning	7.21 ± 2.88	0.866	0.938
6. Social emotional support	6.75 ± 3.22	0.817	0.940
7. Community and services support	7.11 ± 2.89	0.789	0.942
8. Personal fulfilment	7.07 ± 2.88	0.890	0.937
9. Spiritual fulfilment	7.59 ± 2.72	0.673	0.947
10. Global perception of quality of life	7.87 ± 2.69	0.824	0.940

**Table 3:** Factor Loadings for the MQLI-Ar.

Item	Factor Loadings
Physical well-being	0.695
2. Psychological/emotional well-being	0.803
3. Self-care and independent functioning	0.823
4. Occupational functioning	0.813
5. Interpersonal functioning	0.898
6. Social emotional support	0.858
7. Community and services support	0.834
8. Personal fulfilment	0.917
9. Spiritual fulfilment	0.733
10. Global perception of quality of life	0.864

## Data Availability

The data supporting the findings of this study are available from the corresponding author upon reasonable request. Access to the data may be granted for legitimate academic or research purposes, subject to ethical and privacy considerations.
